# A 69-year-old male patient presenting with chest pain and shortness of breath

**DOI:** 10.1007/s12471-019-1259-9

**Published:** 2019-03-12

**Authors:** Y. Saleh, K. Herzallah, M. Elsayed, S. Elkinany, S. Rayamajhi

**Affiliations:** Michigan State University Clinical Center, East Lansing, MI USA

A 69-year-old male patient with a past medical history significant for aortic valve replacement via mechanical valve 35 years ago presented with acute shortness of breath and chest pain.

Upon examination, vital signs were significant for hypotension and tachycardia, lung and heart sounds were unremarkable. Laboratory investigations showed an international normalisation rate (INR) of 3 in addition to mildly elevated D‑dimer, B‑type natriuretic peptide (BNP) and troponin levels. Electrocardiography showed mild ST depression in the lateral leads, chest X‑ray was unremarkable, myocardial perfusion imaging was negative for ischaemia and showed a preserved systolic function. Transthoracic echocardiography and computed tomography were performed (Fig. 1 and Video 1). What is causing his symptoms?

**Fig. 1:**
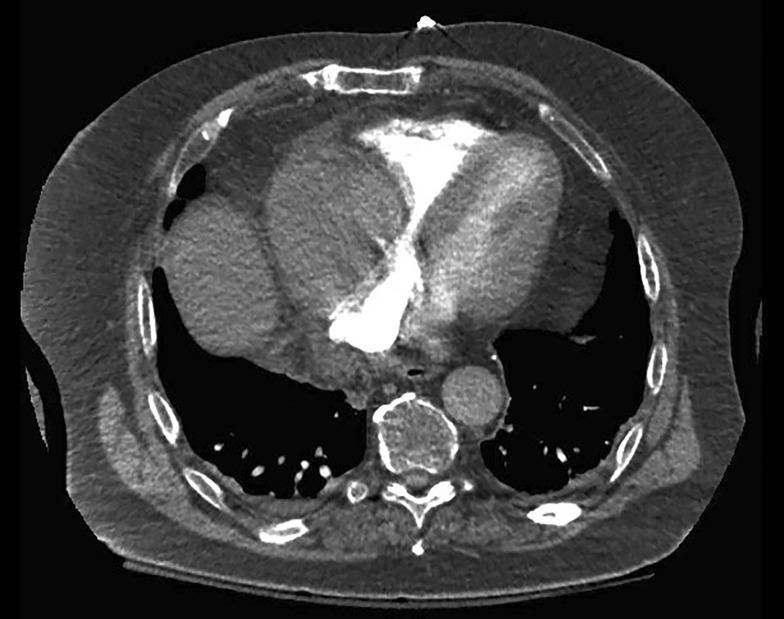
The computed tomography image at presentation

## Answer

You will find the answer elsewhere in this issue.

## Caption Electronic Supplementary Material

Transthoracic echocardiogram in modified parasternal short axis view at the level of the great vessels

